# Characterizing the Impact of the Covid-19 Pandemic on Adults with Autosomal Dominant Polycystic Kidney Disease: A Cross-Sectional Study

**DOI:** 10.21203/rs.3.rs-4406167/v1

**Published:** 2024-06-06

**Authors:** Alok Shetty, Anthony Atalla, Charalett Diggs, Terry Watnick, Stephen Seliger

**Affiliations:** University of Maryland School of Medicine

**Keywords:** ADPKD, Covid-19, Telehealth use, Healthcare avoidance, Chronic disease management

## Abstract

**Background:**

The Covid-19 pandemic greatly affected those with chronic diseases, impacting healthcare access and healthcare seeking behaviors. The impact of the pandemic on adults with Autosomal Dominant Polycystic Kidney Disease (ADPKD) has not been investigated.

**Methods:**

Participants were recruited from a cohort of 239 ADPKD patients enrolled in a longitudinal study at the University of Maryland. Patients on renal replacement therapy were excluded. N = 66 patients participated in a phone questionnaire from June 2022-December 2022 about ADPKD-related complications, concern about contracting Covid-19, healthcare-seeking behaviors, and telehealth utilization before and after March 2020.

**Results:**

N = 34 (51.5%) of participants reported a positive Covid-19 test result and N = 29 (44%) expressed high concern of contracting Covid-19. Those who avoided medical care at least once (N = 17, 25.8%) had similar demographics and ADPKD severity to those who did not, but reported greater telehealth utilization (88.2% vs. 42.9%, p = 0.002), greater use of non-prescribed medication for Covid-19 treatment or prevention (35.3% vs. 8.2%, p = 0.01), and were more likely to contract Covid-19 (76.5% vs. 42.9%, p = 0.02). Among the N = 53 who reported very good or excellent ADPKD disease management pre-pandemic, N = 47(89%) reported no significant change during the pandemic.

**Conclusions:**

In this highly educated, high-income cohort with a mean age of 46.1 years, most people reported well-managed ADPKD prior to the pandemic. This may explain why less than half of participants expressed high concern for contracting Covid-19. Overall, there was no significant pandemic-related decline in self-reported ADPKD management, like due to excellent access to, and uptake of, telehealth services. Notably, 1 in 4 participants reported healthcare avoidant behavior, the effect of which may only be seen years from now. Future studies should investigate potential impacts of avoidant behaviors, as well as expand investigation to a more diverse cohort whose care may not have been as easily transitioned to telehealth.

## Background

Autosomal Dominant Polycystic Kidney Disease (ADPKD) is the most common cause of inherited kidney disease and accounts for approximately 10% of cases of end stage kidney disease (ESKD).^1^ ADPKD progresses with age and typically presents in adulthood.^2^ In addition to intrarenal cysts, patients with ADPKD may also have hepatic cysts, intracranial aneurysms, and cardiac complications.^3^ Blood pressure control is key in the routine management of patients with cystic kidney disease. For patients at high risk of progression, tolvaptan – a V2 receptor antagonist – is the only FDA-approved therapy to slow disease progression, although its use requires frequent safety and laboratory monitoring due to the risk of hepatotoxicity.

The Covid-19 pandemic disrupted healthcare services across the world, leading to modifications in how medical care was delivered. Given their generally high uptake of elective outpatient care, patients with chronic diseases were uniquely impacted. During the early pandemic, there was a greater than 30% decline in ambulatory services, affecting primary and specialty care as well as screening services.^4^ Among US patients with ESRD on chronic dialysis, a substantial proportion experienced difficulties with transportation, medication access, and maintenance of diet during the pandemic.^5^ Additionally, older patients were more likely to have their care disrupted, despite these patients typically needing a higher level of chronic disease management.^6^ More than 40% of chronic disease patients reported their healthcare services were moderately impacted and about one third reported difficulty acquiring their prescribed medications because of the pandemic.^7^ Notably, some patients intentionally avoided perceived high-risk Covid-19 transmission settings such as emergency departments or clinics for symptoms they otherwise would have sought care for in the absence of a pandemic.

As a result of the pandemic, utilization of telehealth services increased, but was only accessible to some due to limiting factors including wireless access and technology literacy. Adequate access to telehealth services proved to be important, as those who were able to utilize telehealth services were less likely to have an emergency department visit or hospital encounter compared to those who were unable to do so.^8^

The degree to which care was disrupted for ADPKD patients because of the Covid-19 pandemic has not been studied. We aimed to investigate how the Covid-19 pandemic impacted self-reported disease management and healthcare-seeking behaviors, including avoidance and telehealth utilization, in a cohort of adult ADPKD patients.

## Methods

We surveyed participants in an ongoing longitudinal observational cohort study (NCT01873235) at the University of Maryland School of Medicine. Participants were at least 18 years of age with ADPKD diagnosed according to the modified Pei-Ravine criteria. Participants with an eGFR of < 15ml/min/1.73m2, receiving dialysis, or with prior kidney transplant were ineligible for participation. Patients provided written informed consent for participation in this ongoing cohort study and had previously agreed to be recontacted for additional data collection and surveys.

This study utilized a telephone-based survey performed from July 2022 to November 2022 among 239 active study participants who remained without ESKD. Patients were contacted by email or phone to inquire about interest in the study. We excluded patients on dialysis or with a prior kidney transplant. If participants agreed to the survey, it was conducted at that time over the phone, or a call was scheduled later to conduct the survey. Study team members explained the purpose and implications of the study, answered patients' questions, and obtained verbal consent. Patients were informed they could opt out of answering any of the survey items. Patient identifiers were confirmed before starting the survey. Based on a questionnaire from Splinter et al., the survey consisted of 44 items covering participant demographics, pre and post pandemic health status and healthcare utilization, Covid-19 infections, concern of contracting Covid-19, medication use and access, and lifestyle modifications.^9^ The pre-pandemic period was identified as prior to March 2020 and post-pandemic was identified as after March 2020. The Institutional Review Board at the University of Maryland School of Medicine approved this study (protocol HP-00054815) and waived the requirement for written documentation consent for the questionnaire. The questionnaire used in this study is provided in the supplementary information.

### Assessments:

Participants received laboratory testing, vital sign measurement, renal imaging and comprehensive medical history including major ADPKD-related complications at initial enrollment and after 3 years; clinical information collected at the visit closest in time to the Covid-19 telephone survey was used for this analysis. GFR was estimated from serum creatinine using the CKD-Epi estimating equation.^10^ Patients without contraindications received abdominal MRI and total kidney volume indexed to height was estimated as previously described.^11^

The primary outcomes of this study were a patient’s concern of contracting Covid-19, and self-reported ADPKD management before and after the pandemic. To assess Covid-19 concern, we asked: “How often did you feel concerned about contracting Covid-19 infection?” Response choices included (i) never, (ii) rarely, (iii) sometimes, (iv) often, and (v) almost continuously. Related to Covid-19 concern, we also asked “To what extent did having ADPKD contribute to your concern about contracting Covid-19 infection?” Response choices included (i) not at all, (ii) somewhat, (iii) moderately, (iv) a lot.

To assess self-reported ADPKD management, participants were asked: “How well did you feel your ADPKD was managed prior to the Covid-19 pandemic?” and “How well did you feel your ADPKD was managed since the start of the Covid-19 pandemic?” Response choices included (i) excellent, (ii) very good, (iii) good, (iv) fair, and (v) poor.

Additionally, we assessed the avoidance of healthcare settings during the pandemic and telehealth usage. To assess “care avoidance,” participants were asked “Since the start of the Covid-19 pandemic, how many times have you avoided seeking medical care for a symptom or condition that you otherwise would have sought medical attention for?” Response choices included (i) never, (ii), once, (iii) fewer than five occasions, and (iv) more than five occasions. To assess telehealth utilization, participants were asked “Since March 2020, how many times have you utilized telehealth (telephonic, video) healthcare services?” Participants were asked to provide numerical responses.

### Statistical Analysis:

“High concern” was characterized as a response of almost continuously or often. “low concern” was characterized as a response of sometimes, rarely, or never. “Well managed disease” was defined as a response of excellent or very good and “poorly managed disease” was defined as a response of good, fair, or poor. Participants who were classified as having experienced “care avoidance” were those who responded once, fewer than five occasions, or more than five occasions and only those who responded never were classified as having “no care avoidance.” Participants who utilized telehealth on 5 or more occasions were included in the group of “High telehealth utilization” and those who utilized telehealth on less than 5 occasions were included in the “Low telehealth utilization” group.

Differences in characteristics between subgroups were compared using Fisher’s Exact Chi-squared tests and independent sample t-tests. Four individuals were diagnosed with ADPKD after March 2020, so they were excluded from the analysis comparing pre-pandemic vs post-pandemic ADPKD disease management. Findings were deemed significant with a p-value ≤ 0.05. All statistical analyses were conducted using Stata 17 (Statacorp, College Station TX).

## Results

We invited N = 194 ADPKD patients participating in the ongoing observational study to participate in this Covid-19 survey. Of these, N = 69 agreed to participate and provided responses to survey questions over the phone (overall response rate, 36%). However, 3 of these participants were excluded from final analysis as they had developed ESRD requiring dialysis or kidney transplantation prior to the survey, resulting in a final sample size of 66. Table 1 compares characteristics amongst both participants (i.e. those who responded to the present study questionnaire) and non-responders. Age, sex, and race, eGFR, htTKV, and reported ADPKD-related complications and comorbidities did not differ significantly between responders and non-responders, apart from hypertension, which was modestly more common among non-participants. Among responders who participated in the questionnaire, mean (SD) age was 46.1 (13.3) years, 51% were female, 91% were of self-reported White race/ethnicity, 83% had at least a college education and mean (SD) eGFR was 75.2 (32.2) ml/min/1.73m2, with 25 (37.9%) having an eGFR < 60ml/min/1.73m2.

Concern about contracting Covid-19: A total of N = 34 (51.5%) of participants reported a positive Covid-19 test result sometime during the pandemic; of these, N = 5 (14.7%) had more than one episode of infection. Only N = 2 participants required inpatient care for Covid-19. N = 62 (93.4%) of participants reported being fully vaccinated at the time of the survey. Table 2 compares characteristics between participants who expressed high concern about contracting Covid-19 (N = 29, 44%) to those who expressed low concern (N = 37, 56%). Participants with high concern vs. low concern did not differ with regards to demographic factors, educational achievement, ADPKD severity, or frequency of major ADPKD-related complications. In addition, the two groups did not differ with respect to frequency of Covid-19 infection, healthcare avoidance due to Covid-19, telehealth utilization, and use of non-prescribed medications to prevent Covid-19 infection.

Healthcare avoidance during the Covid-19 pandemic: Comparing those who reported new healthcare avoidance during the pandemic (N = 17, 25.8%) to those without healthcare avoidance (N = 49, 74.2%), those with healthcare avoidance endorsed higher rates of Covid-19 infection [76.5% vs. 42.9% (p = 0.02)], increased use of non-prescribed medications to treat or prevent Covid-19 [35.3% vs. 8.2% (p = 0.01)], and were more likely to report “high” levels of telehealth utilization [88.2% vs. 42.9% (p = 0.002)] (Table 3). Those with new healthcare avoidance did not express higher levels of concern about contracting Covid-19. Furthermore, those with healthcare avoidance were not significantly different with regards to ADPKD severity, ADPKD complications, or demographics.

**Figure 1:**
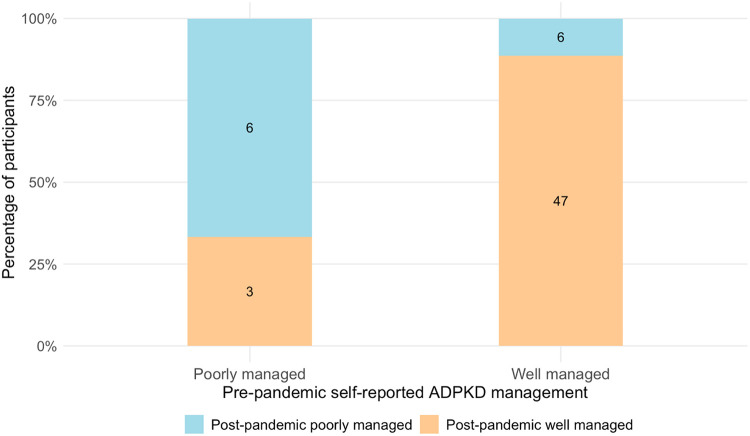
Participants’ self-reported polycystic kidney disease management before and after the start of the Covid-19 pandemic. The x-axis indicates the participants’ pre-pandemic assessments and the proportion of individuals who changed or maintained their response is indicated by the vertical bars. PKD: polycystic kidney disease

**Figure 2:**
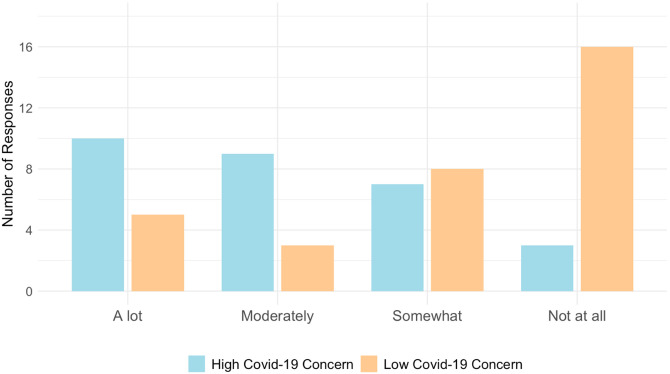
Participants’ response to “To what extent did having polycystic kidney disease contribute to your concern of contracting Covid-19?” compared between those determined to have “high” or “low” concern of contracting Covid-19. ADPKD: Autosomal Dominant Polycystic Kidney Disease.

Perception of ADPKD disease during the Covid-19 pandemic: Among those who reported either “very good” or “excellent” ADPKD disease management prior to the pandemic (N = 53, 80.3%), N = 47 (89%) reported no significant change in their ADPKD self-management after the pandemic began (Fig. 1). Those with worse self-reported disease management prior to the pandemic did not report increased concern about contracting Covid-19 in our survey. However, as noted in Fig. 2, those who expressed increased concern about contracting Covid-19 in general attributed more of that concern to their ADPKD diagnosis (p = 0.003).

## Discussion

We examined attitudes, concerns, and healthcare behaviors and practices in response to the Covid-19 pandemic among 66 adult patients with ADPKD from Maryland and the Mid-Atlantic US. Our aim was to characterize these patients’ lived experience during the pandemic, with potential implications for others living with other chronic organ-threatening diseases impacted by Covid-19, and with implications for their care in any future pandemic or public health emergency.

There are a few key findings from this study that we wish to highlight. First, patients in this cohort who stated that their ADPKD was “well-managed” prior to the pandemic overwhelmingly reported minimal decline in their self-reported disease status after the pandemic began. This differs from previously published literature regarding progression and worsening prognosis of other diseases, such as diabetes and hypertension, as a result of pandemic-related barriers to healthcare access and disease surveillance. ^12,13^ We hypothesize a few reasons for these observed differences. Generally, patients in this cohort had adequate access to telehealth services throughout the pandemic, reporting an average of 4.39 telehealth appointments. This may have enabled them to continue regular disease surveillance and management despite not being able to access in-person care, especially during the early phase of the pandemic. Additionally, disease management was ascertained in our study by self-report, which is inherently subjective. Monitoring of ADPKD progression tends to be based on objective metrics, such as blood pressure and eGFR, and imaging, such as htTKV. ^14,15^ As such, patients who may not have noticed a change in physical symptoms or were unaware of worsening objective measures of disease progression may have reported their disease as “well-managed” despite objective disease progression that may not occur until years into the future.

Second, those who were more concerned about contracting Covid-19 did not appear to differ significantly in our metrics of disease severity (htTKV, eGFR, and complication rates prior to the pandemic). This lack of difference was unexpected, as it might be expected that those with more advanced disease would have greater concern for contracting Covid-19 due to the potentially more severe consequences of infection among these patients. However, those who expressed more concern about contracting Covid-19 did attribute more of this concern to their ADPKD diagnosis than those who were had less concern about contracting Covid-19. This discrepancy could be attributed to participants having different levels of fear and concern related to their health, irrespective of the objective severity of their ADPKD disease. In fact, Mertens et al. explored the topic of “fear” of the coronavirus, and found four main predictors of increased fear of, or concern about, contracting the virus, one of which was general health anxiety.^16^ Overall, just 29 of the 66 participants in our study expressed “high” concern about contracting Covid-19, which is notable considering prior reports of overall higher rates of stress and anxiety about the virus in those with comorbid conditions compared to their healthier counterparts.^17^ In general, it does not appear that this cohort of ADPKD patients were particularly concerned about contracting Covid-19, which could be attributed to their relatively younger mean age (46.1 years) and overall well-managed ADPKD disease prior to the pandemic, with a cohort median htTKV of 694.31 and mean eGFR of 75.2. Further studies of Covid-19 influenced health behaviors in ADPKD patients without access to specialized ADPKD-related care, older age, and more advanced disease are warranted.

Lastly, 17 participants (25%) in our study reported avoiding in-person medical care during the pandemic on one or more occasions. Studies surveying care avoidance during the pandemic in the general population have demonstrated rates between 8% and 41%.^18^ A phenomenon documented in both acute and routine care settings, it has been linked to a variety of factors such as younger age, inability to afford care, and greater Covid-19-related stress.^19-21^ In our study, we found no association between participant concern about contracting Covid-19 or ADPKD disease severity and in-person healthcare avoidance. However, participants who avoided in-person care had higher rates of Covid-19 infection, reported greater use of non-prescribed medications and supplements to treat and/or prevent Covid-19, and were more likely to report “high” levels of telehealth use. The increased uptake of telehealth services in this generally high-income, well-educated group makes sense, as it was a viable option for those who did not wish to enter in-person clinical settings. However, it is still notable that 1 in 4 participants avoided in-person care in some capacity. While telehealth can be a useful tool in surveillance of chronic disease, early descriptive studies out of the pandemic have demonstrated worsening of certain pre-existing chronic issues, such as chronic pain, primarily as a result of in-person appointment cancellations and postponements.^22^ Considering that vital aspects of patient care and disease surveillance are lost when in-person evaluation cannot occur, the implications of this avoidance on long-term disease progression are uncertain and could manifest in the coming years. This pattern of care avoidance could also be found and is especially more concerning amongst patients with chronic conditions where disease progression manifests on a more rapid timescale than ADPKD.

There are a few limitations in this study that we wish to address. The ordinal response system that many of our questions used is subject to recall bias and can be subject to varying interpretations by respondents. In addition, the vast majority of patients in this cohort identified as White, college-educated, and reported annual incomes of over $100,000. The demographic characteristics of our participants largely mirror those of the greater observational cohort study from which we drew, which was 80.6% White and 84.6% college-educated at the time of our survey. This lack of racial and socioeconomic diversity means that a large portion of ADPKD patients who do not fit these demographics are not represented. Existing literature suggests the disproportionate effects the Covid-19 pandemic had on progression of other chronic diseases, as well as morbidity and mortality related to chronic issues such as hypertension and chronic kidney disease.^23,24^ As such, future studies should strive to include patients from a variety of demographic groups in order to represent the unique set of circumstances they face regarding their ADPKD disease management and healthcare access. Another limitation of this study is a relatively small sample size, which led to even smaller sizes of stratification groups. Expanding this study to include ADPKD patients from centers across the country would expand the sample size, increase the validity of the results, and address the limitations in geographic, socioeconomic, and racial diversity.

## Conclusions

In summary, ADPKD patients in this well-educated, health-literate population did not experience a significant worsening in their self-reported ADPKD-related disease management during the Covid-19 pandemic. They reported overall only modest levels of concern about contracting Covid-19. However, 1 in 4 participants in this cohort reported avoidance of in-person care during the avoidance during the pandemic. As mentioned above, the implications of this avoidance on long-term disease prognosis are unknown. However, it appears that the pandemic did not significantly alter short-term ADPKD disease prognosis in most of these patients. This may be attributable to participants’ widespread access to quality telehealth care throughout the pandemic, indicating the value that telehealth care models may have in future settings of restricted access to in-person care. However, this study also highlights the demographic homogeneity in those represented in ADPKD research and demonstrates the importance of increasing diversity in patient populations used for future related research.

## Figures and Tables

**Table 1:** Characteristics of ADPKD patients who participated compared to those who did notparticipate in Covid-19 questionnaire.

Demographic Characteristic	Participated (n = 66)	Did not participate (n = 157)	p-value
Age (years)	46.1 (13.3)	46.8 (14.7)	p = 0.71
**Race**			
White	60 (90.9%)	117 (74.5%)	p = 0.06
Non-White	6 (9.1%)	39 (24.8%)	
**Sex**			
Female	37 (56.1%)	101 (64.3%)	p = 0.22
Male	29 (43.9%)	55 (35.0%)	
College Education	55 (83.3%)	119 (75.8%)	p = 0.15
Mean eGFR (mL/min/1.73m2)	75.2 (32.2)	72.1 (32.5)	p = 0.51
Median htTKV (mL/m)	694.3 [452.9, 1275.1]	843.7 [537.2, 1549.3]	p = 0.14
**ADPKD Complications**			
Hypertension	40 (60.6%)	116 (73.9%)	p = 0.03*
Flank pain	30 (45.5%)	89 (56.7%)	p = 0.09
Kidney stones	10 (15.2%)	19 (12.1%)	p = 0.56
Urinary tract infection	36 (54.6%)	84 (53.5%)	p = 0.97

**Table 2:** Characteristics of participants who expressed high Covid-19 concern compared to those with low Covid-19 concern

Demographic Characteristic	High Covid Concern (n = 29)	Low Covid Concern (n = 37)	p- value
Age (yr)	44.1 (13.5)	47.6 (13.2)	p = 0.29
**Race**			
White	25 (86.2%)	35 (94.6%)	p = 0.25
Non-White	4 (13.8%)	2 (5.4%)	
**Sex**			
Female	18 (62.1%)	19 (51.4%)	p = 0.38
Male	11 (37.9%)	18 (48.6%)	
College Education	22 (75.9%)	33 (89.2%)	p = 0.15
Annual Income ≥ $60,000	26 (92.9%) *1 declined to answer*	33 (97.1%) *3 declined to answer*	p = 0.48
Mean eGFR (mL/min/1.73m2)	73.1 (33.4)	76.9 (31.6)	p = 0.63
Median htTKV (mL/m)	642.1 [427.0, 1162.8]	838.6 [467.0, 1350.1]	p = 0.38
**ADPKD Complications**			
Hypertension	19 (65.5%)	21 (56.8%)	p = 0.47
Flank pain	11 (37.9%)	19 (51.4%)	p = 0.28
Kidney stones	4 (13.8%)	6 (16.2%)	p = 0.89
Urinary tract infection	18 (62.1%)	18 (48.7%)	p = 0.25
**Covid-19 pandemic-related health behaviors**			
New care avoidance during pandemic	8 (27.6%)	21 (56.8%)	p = 0.78
Use of non-prescribed medications for Covid-19 prevention/tx	5 (17.2%)	5 (13.5%)	p = 0.74
Covid-19 infection during pandemic	15 (51.7%)	19 (51.4%)	p = 1.00
“High “telehealth use	15 (51.7%)	21 (56.8%)	p = 0.80

**Table 3:** Characteristics of participants with and without new avoidance of healthcare during Covid-19 pandemic

Demographic Characteristic	No avoidance (n = 49)	Avoidance (n = 17)	p-value
Age	45.5 (14.0)	47.8 (11.4)	p = 0.54
Male	22 (44.9%)	7 (41.2%)	p = 1.00
White	44 (89.8%)	16 (94.1%)	p = 1.00
College education	40 (81.6%)	15 (88.2%)	p = 0.72
Annual Income ≥ $60,000	43 (93.5%) *3 declined to answer*	16 (100%) *1 declined to answer*	p = 0.31
eGFR (ml/min/1.73m2)	77.9 (32.4)	67.6 (31.2)	p = 0.26
htTKV (cc/m)	669.7 [439.4, 1279.0]	838.6 [658.2, 1108.9]	p = 0.30
**ADPKD Complications**			
Hypertension	30 (61.2%)	10 (58.8%)	p = 0.86
Flank Pain	21 (42.8%)	9 (52.9%)	p = 0.58
Kidney Stones	6 (12.8%)	4 (23.5%)	p = 0.20
UTIs	24 (49.0%)	12 (70.6%)	p = 0.25
**Covid-19 pandemic related health behaviors**			
Concern about contracting Covid	21 (42.9%)	8 (47.0%)	p = 0.78
Use of non-prescribed medications for Covid prevention/tx	4 (8.2%)	6 (35.3%)	p = 0.01*
Covid-19 infection during pandemic	21 (42.9%)	13 (76.5%)	p = 0.02*
“High” telehealth use	21 (42.9%)	15 (88.2%)	p = 0.002*

## Abbreviations

ADPKD: Autosomal Dominant Polycystic Kidney Disease
Covid-19: Coronavirus Disease 2019
eGFR: Estimated glomerular filtration rate
ESKD: End-stage kidney disease
htTKV: Height-adjusted total kidney volume
